# Treatment of Hygromain in the Front Knees of Cattle

**Published:** 1897-01

**Authors:** 


Treatment of Hygromain in the Front Knees of Cattle. Proussier had an extended experience with this affection while he was practising in the mountain district of Père. After having tried the different common modes of treatment, such as opening the sac along its whole length, applying iodine solutions, etc., without any result, he made a small incision at the lowest point of swelling, and drew from the top to the bottom through the whole cavity a string saturated with tincture of iodine. The upper surface of the swelling he had rubbed very thoroughly. During the next few days the knee was much swollen. After that the swelling decreased from day to day. The opening in the sac was treated with antiseptic applications. The string remained, as a rule, twenty-five to thirty days, which was usually sufficient to bring about a cure.—*Schweizer Archiv für Tierheilkunde*, January and February, 1896.

